# Effects of N-Terminal and C-Terminal Polyhistidine Tag on the Stability and Function of the Thermophilic P450 CYP119

**DOI:** 10.1155/2019/8080697

**Published:** 2019-06-20

**Authors:** Yaprak Aslantas, Nur Basak Surmeli

**Affiliations:** ^1^Program in Biotechnology and Bioengineering, İzmir Institute of Technology, İzmir, Turkey; ^2^Department of Bioengineering, İzmir Institute of Technology, İzmir, Turkey

## Abstract

Biocatalysts are sought-after in synthesis of pharmaceuticals and agrochemicals due to their high regioselectivity and enantioselectivity. Among biocatalysts, heme-containing cytochrome P450 (P450) oxygenases are an attractive target since they catalyze oxidation of “unactivated” carbon-hydrogen bonds with high efficiency. CYP119 is an acidothermophilic P450 from *Sulfolobus acidocaldarius*, which has the potential to be widely used as a biocatalyst since it shows activity at high temperatures and low pH. Polyhistidine tags (His-tags) are widely used to simplify purification of proteins. However, His-tags can cause changes to protein structure and function. Here, we demonstrate the effects of His-tags on CYP119. To this end, the His-tags were cloned at the N-terminus or C-terminus of the CYP119, and His-tagged proteins were expressed and isolated. The thermostability and peroxidase activity of His-tagged CYP119s were tested and compared to wild type CYP119. Results indicated that while addition of His-tags increased the yield and simplified isolation of CYP119, they also influenced the electronic structure of active site and the activity of the protein. We show that N-terminal His-tagged CYP119 has desirable properties and potential to be used in industrial applications, but mechanistic studies using this protein need careful interpretation since the His-tag affects electronic properties of the active site heme iron.

## 1. Introduction

Enzymes catalyze reactions with high regioselectivity and enantioselectivity. These key features of biocatalysts, i.e., enzymes, result in their increased utilization in the synthesis of pharmaceuticals and agrochemicals. Amongst biocatalysts, heme-containing cytochrome P450 (P450) oxygenases are a particularly attractive target because of their capability to catalyze oxidation of hydrocarbons with high efficiency and selectivity [1–3]. Indeed, P450s are currently used in pharmaceutical industry in the synthesis of drugs such as progesterone used in the treatment of uterine and cervical cancer and cortisone used in treatment of allergy and inflammation [4]. In nature, P450s are involved in the hydroxylation of hydrophobic substrates [5] and participate in the metabolism of xenobiotics and biosynthesis of critical signaling molecules [6].

Among P450s, thermophilic ones have the most potential applications since thermophilic enzymes are more stable towards mutations, high temperature, high and low pH, and organic solvents. Therefore, the enzyme CYP119 from the acidothermophilic archaea *Sulfolobus acidocaldarius* is a very appealing target [7–9]. *S. acidocaldarius* has an optimum growth temperature of 83°C and 2.4 pH [10]. CYP119 is one of the most extensively studied thermostable P450s, and its molecular structure was determined by X-ray crystallography [2]. Currently, the native substrate for CYP119 is not known; however, CYP119 can catalyze epoxidation of styrene and the oxidation of *N*-acetyl-3,7-dihydroxyphenoxazine (Amplex Red) by H_2_O_2_. One of the limitations in the practical application of CYP119 in the industry is that the expression and isolation of native CYP119 is tedious and time consuming [11]. These problems can be solved by incorporation of polyhistidine tags (His-tags) to facilitate isolation of the protein.

His-tags, which typically contain consecutive six or more histidine residues, are now commonly used in protein purification. His-tagged proteins are easily separated from the cell lysate by using immobilized metal affinity chromatography (IMAC). In IMAC, divalent cations (usually Ni^2+^ or Co^2+^) are adhered to a solid matrix; around neutral pH, the histidine residues form complexes with the chelated metal ions. The protein of interest can then be eluted by displacement of histidine by addition of imidazole to the mobile phase or changing the pH of the solution. Since His-tags are relatively small in size (∼2.5 kDa), they are thought to not affect the structure and function of proteins [12]. Yet, recent reports suggest that this assumption can be incorrect [13–15]. While the application of His-tags for protein purification has been extremely useful in increasing purity and yield of recombinant proteins, disregarding the consequences of His-tags on native protein structure and function will lead to nonreproducibility and confusion in the literature [16].

Here, the effects of N-terminal and C-terminal His-tags on the structure function and stability of the enzyme CYP119 were investigated. CYP119 was expressed and purified with N-terminal and C-terminal His-tags (N-His-CYP119 and C-His-CYP119). The thermostability of the proteins obtained was characterized. In addition, peroxidase activity of these enzymes and have been carried out at different temperatures. N-His-CYP119 showed highest activity and stability under the conditions tested, so the kinetic parameters of this protein in Amplex Red oxidation with H_2_O_2_ were also investigated.

## 2. Materials and Methods

### 2.1. Cloning of His-Tagged CYP119

The CYP119 gene in pET11a plasmid was a gift from Teruyuki Nagamune (Addgene, #66131) [17]; it was cloned to plasmids containing Hexa-histidine tag. The N-terminal His-tag was cloned with *NdeI* and *BamHI* restriction enzymes to pET-14b. C-terminal His-tag was cloned with *XbaI* and *BamHI* restriction enzymes to pET-20b (+). C-terminal His-tag addition required mutagenesis to delete the stop codon between the His-tag and the CYP119 sequence. This was accomplished with polymerase chain reaction (PCR) using Q5 site directed mutagenesis kit (BioLabs). The designed primers used for PCR were TCCGTCGACAAGCTTGCG and TTCATTACTCTTCAACCTGACCAC.

### 2.2. Expression of His-Tagged CYP119s

The plasmids containing His-tagged CYP119s were transformed to *E. coli* BL21 (DE3) expression cells. Recombinant *E. coli* BL21 (DE3) was cultured in 10 mL of Lysogeny Broth (LB, 10 g/L tryptone, 5 g/L yeast extract and 10 g/L NaCl) with 0.1 mg/mL ampicillin. The culture was incubated overnight at 37°C in 220 rpm shaking incubator. Overnight culture (4 mL) was inoculated to 500 mL of 2xYT Medium Broth (16 g/L tryptone, 10 g/L yeast extract and 5 g/L NaCl at pH 6.8) with 0.1 mg/mL ampicillin and grown at 37°C with shaking until optical densities reached 0.8 at OD_600._ Expression of C-His-CYP119 was induced with 1 mM Isopropyl ß-D-1-thiogalactopyranoside (IPTG) and 0.5 mM *δ*-aminolevulinic acid (ALA) for 16 hrs at 30°C. The N-His-CYP119 expression was induced with 0.5 mM IPTG and 0.3 mM ALA for 8 hrs at 30°C. The cells were harvested by centrifugation at 3800 rpm for 20 minutes. The cell pellet was kept at −80°C until purification.

### 2.3. Isolation and Purification of His-Tagged CYP119s

The frozen cell pellets obtained were dissolved in lysis buffer (150 mM NaCl, 10 mM imidazole, 0.2 mM phenylmethylsulfonyl fluoride (PMSF), 1 mM benzamidine HCl, 50 mM potassium phosphate at pH 7.5). Cells were ultrasonicated, and cell lysates were incubated at 65°C for 1 h, followed by centrifugation 3900 rpm for 2 hours at 4°C. For WT CYP119, the supernatant was used as is or enriched by ammonium sulfate (60%) precipitation. WT CYP119/ammonium sulfate pellet was resuspended in potassium phosphate buffer (50 mM) at pH 7.5 and dialyzed against the same buffer. For His-tagged CYP119, after heat treatment, the supernatant was passed through a Ni-NTA column (Affinity, Agarose Resin, Thermo Scientific™) and washed with potassium phosphate buffer (50 mM) containing NaCl (150 mM), imidazole (10 mM), PMSF (0.2 mM), and benzamidine HCl (1 mM) at pH 7.5. CYP119 was eluted from the column with imidazole (150 mM), and NaCl (20 mM) in potassium phosphate buffer (50 mM) at pH 7.5. The fractions containing His-tagged CYP119 (47 kDa) were pooled and buffer-exchanged to potassium phosphate (50 mM) at pH 7.5 with a desalting column (Thermo Scientific) with a molecular cutoff of 10 kDa. 50 *μ*l samples from every purification step were taken to be analyzed by SDS-PAGE. The protein purity level was determined using GelAnalyzer (http://www.gelanalyzer.com) program. CYP119 protein concentrations were determined with the known extinction coefficient (*ε*415 nm = 104 mM^−1^ cm^−1^) [1].

### 2.4. UV-Visible Spectra of CYP119s

The UV-Visible spectra of WT and His-tagged CYP119 proteins were obtained in 50 mM potassium phosphate buffer at pH 7.5. UV-Visible spectrophotometric analysis was carried out using a UV-1600PC Scanning Spectrophotometer for the WT and His-tagged CYP119. The spectra were deconvoluted with fityk 1.3.1 software by fitting to Gaussian functions with Levenberg–Marquardt method after baseline subtraction [18].

### 2.5. Thermostability Measurements of CYP119s

To test thermostability, the enzymes were incubated for 5 minutes at increasing temperatures (25, 60, 70, 75, 80, 85, 90, and 95°C) in 50 mM potassium phosphate buffer at pH 7.4. UV-visible spectra were taken at each temperature between wavelengths 350–650 nm. The difference spectra were obtained by subtraction of the spectrum at room temperature from the spectra obtained at higher temperatures. The shift in maximum Soret absorbance was monitored by ΔΔAbs (390–425), where ΔAbs is defined by the difference of absorbance at the specified wavelength at higher temperature from the absorbance at room temperature. ΔΔAbs (390–425) is obtained by subtraction of ΔAbs_390nm_ from ΔAbs_425nm_.

### 2.6. Peroxidase Activity of CYP119s

The peroxidase activity of WT CYP119, N-His-CYP119, and C-His-CYP119 was determined by Amplex Red (Thermo Fisher Scientific) oxidation reactions. The reactions contained 10 *μ*M Amplex Red, 1.5 mM H_2_O_2_, and 1.5 *μ*M CYP119 variants in 0.25 M sodium phosphate at pH 7.4. The reactions were performed at room temperature and 65°C. Formation of the product (resorufin) was followed by monitoring of fluorescence (570 nm excitation, 585 nm emission) or absorbance at 570 nm (*ε*_570nm_ = 54 mM^−1^·cm^−1^) [6]. Negative control was performed without H_2_O_2_. The average of the emission values of the resorufin at 582 nm from three independent measurements was used. Observed rate constant (*k*_obs_) was obtained by fitting kinetic data to one phase association equation.

### 2.7. Kinetic Analysis of Amplex Red Oxidation by N-His-CYP119

Kinetic analysis of the Amplex Red reaction was followed by UV-Visible Spectroscopy. The reaction was performed with 10 *μ*M Amplex Red, 1.5 mM H_2_O_2_ and 1.5 *μ*M N-His-CYP119 in 50 mM reaction buffer at pH 7.4. UV-Visible spectra were taken at 0, 2, 5, 10, 20, 30, 45, 60, 90, 120, 150, 180, and 270 minutes after addition of H_2_O_2_. To determine the kinetic parameters for Amplex Red oxidation by N-His-CYP119, formation of resorufin was followed at 570 nm during the reaction of Amplex Red (10 *μ*M) with H_2_O_2_ (0.25–2 mM) in the presence of N-His-CYP119 (1.5 *μ*M). The initial rate data was fit to the Michaelis–Menten equation to obtain the kinetic constants.

## 3. Results

### 3.1. Cloning Expression and Purification of His-Tagged CYP119

**Figure 1:**
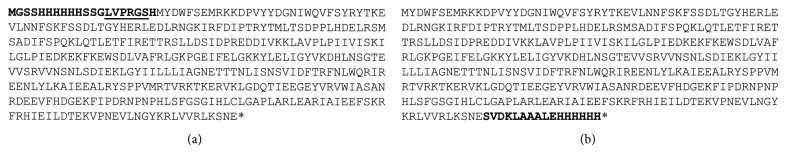
Amino acid sequences of CYP119s cloned with an N-terminal (a) and C-terminal (b) His-Tag. The roman characters show the sequence of WT CYP119, and the bold amino acids are additional residues cloned. N-terminal His-tagged CYP119 also includes a thrombin cleavage site which is underlined.

The sequences of the proteins N-His-CYP119 and C-His-CYP119 are shown in Figure 1, the additional residues are shown in bold. Both proteins were cloned and expressed *E. coli* as described in the materials and methods section. Purification involved initial heat treatment of cell lysate at 65°C for 1 hr. As seen in Figure S1, all proteins were still highly soluble after heat treatment. This was followed by IMAC, which led to approximately 95% purity for both proteins. From 0.5 L of culture 4.8 mg of N-His-CYP119 and 1.4 mg of C-His-CYP119 were obtained. The heme incorporation of isolated proteins were observed by the absorbance ratio of 280 nm vs Soret (415 nm) absorbance, this ratio was 0.46 and 0.58 for N-His-CYP119 and C-His-CYP119, respectively, showing appropriate levels of heme incorporation for both proteins.

### 3.2. Electronic Absorption Spectroscopy of WT CYP119, N-His-CYP119, and C-His-CYP119

**Figure 2:**
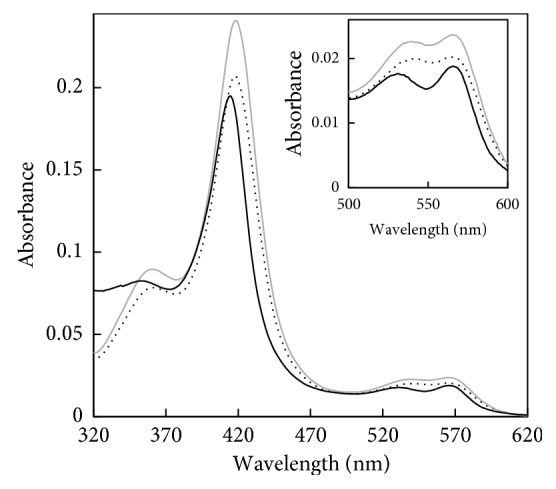
The comparison of UV-Visible spectra of C-His-CYP119 (dash) and N-His-CYP119 (grey) with WT CYP119 (black). Inset: the changes observed in the *α*/*β* bands.

The optical spectra of N-His-CYP119, C-His-CYP119, as isolated, and enriched WT CYP119 are shown in Figure 2. In order to get a more accurate comparison of the absorbance maxima, the Soret and *α*/*β* bands were deconvoluted and maximum absorbances were obtained from the deconvoluted spectra. The fit of deconvoluted peaks is shown in (Figures S2 and S3). After deconvolution, WT CYP119 shows maximum Soret absorbance at 414 nm and split *α*/*β* bands in 533 and 567 nm, similar to previously reported spectra (Figure S2) [3]. Addition of the His-tag to N-terminal and C-terminal of the protein resulted in a small shift in the Soret maximum to 418 nm (Figure S3). The *α*/*β* bands shifted to 535 and 570 nm for C-His-CYP119 and to 540 and 572 nm for N-His-CYP119 (Figure 2, Figure S3).

### 3.3. Thermostability Measurements of WT CYP119, N-His-CYP119, and C-His-CYP119

**Figure 3:**
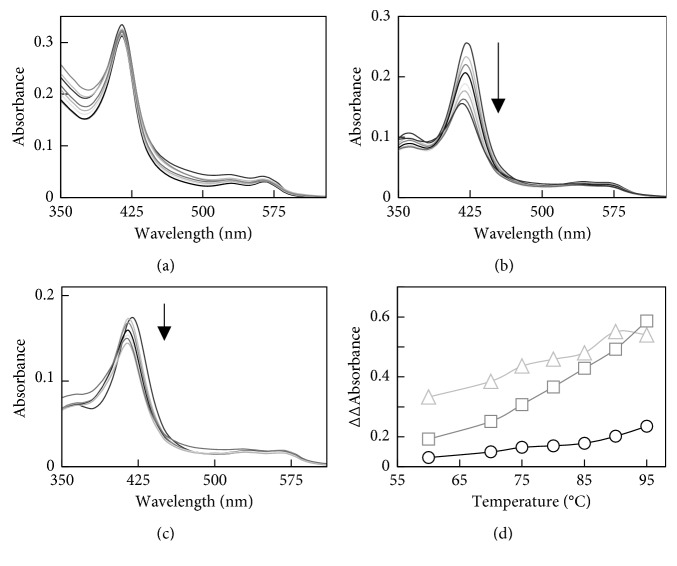
UV-Visible spectra of thermostability analysis of 3.5 *μ*M WT CYP119 (a), 2 *μ*M N-His-CYP119 (b), and 1.4 *μ*M C-His-CYP119 (c). Spectra were taken at different temperatures (25, 60, 70, 75, 80, 85, 90, and 95°C) after incubation for 5 minutes in 50 mM potassium phosphate buffer at pH 7.4. The normalized ∆∆Abs (390–425) for C-His-CYP119 (△) and N-His-CYP119 (□) and WT CYP119 (◯) with respect to the temperature increase from 25°C to 95°C (d).

Changes in the UV-Visible spectra of N-His-CYP119, C-His-CYP119, and WT CYP119 with temperature are shown in Figure 3. The maximum Soret absorbance of WT CYP119 does not change with temperature up to the highest temperature tested (95°C) (Figure 3(a)). However, there is an increase in absorbance at lower wavelengths at higher temperatures due to aggregation caused by impurities. On the other hand, maximum Soret absorbance of N-His-CYP119 and C-His-CYP119 shifted from 418 nm to 415 nm with increasing temperature (Figures 3(b) and 3(c)). In addition, the Soret absorbance of N-His-CYP119 and C-His-CYP119 decreased by 40% and 12%, respectively, with increase in temperature (Figures 3(b) and 3(c)). Difference spectra can be used to closely monitor the changes in the Soret absorbance with temperature (Figure S4). The difference spectra show that, for His-tagged CYP119s, the maximum decrease in absorbance is observed at 425 nm and a subtle increase in absorbance at 390 nm accompanies the decrease in 425 nm (Figure S4B). This shift in absorbance can best be monitored by the change in the difference in absorbance at 390 nm and 425 nm [∆∆Abs (390–425)]. As seen in Figure 3(d), the shift in absorbance [∆∆Abs (390–425)] is highest for N-His-CYP119, while no significant shift is observed for WT CYP119.

### 3.4. Peroxidase Activity of WT CYP119, N-His-CYP119, and C-His-CYP119

**Figure 4:**
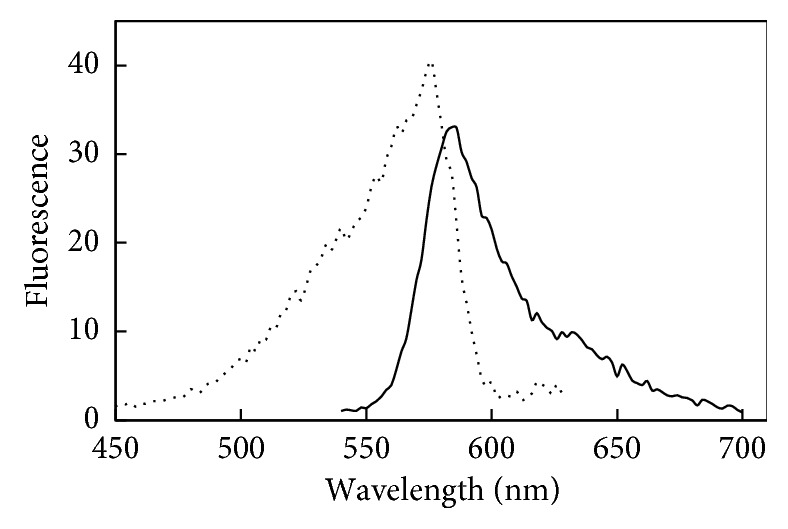
The excitation (solid) and emission (dash) fluorescence of the Amplex Red oxidation product (resorufin) catalyzed by WT CYP119. Reaction components were 10 *μ*M Amplex Red, 1.5 mM H_2_O_2_, and 1.5 *μ*M WT CYP119 in 50 mM sodium phosphate at room temperature at pH 7.4, spectra taken after 40 minutes.

The peroxidase activity of CYP119 variants was investigated by following oxidation of Amplex Red by H_2_O_2_ [9]. The product resorufin can be observed by fluorescence with excitation at 570 nm and emission at 585 nm. The excitation and emission spectra of the reaction mixture containing 1.5 mM H_2_O_2_ and 10 *μ*M Amplex Red with 1.5 *μ*M WT CYP119 is shown in Figure 4, the spectra clearly show resorufin formation.

**Figure 5:**
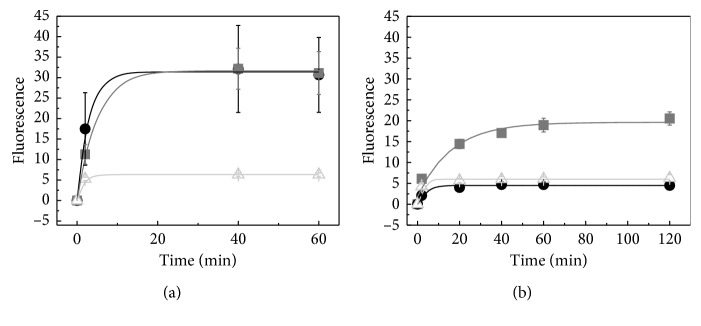
Kinetic analysis of Amplex Red reaction with WT CYP119 (•), N-His-CYP119 (■), and C-His-CYP119 (△) at room temperature (a) and 65°C (b). Reactions were performed using 10 *μ*M Amplex Red, 1.5 mM H_2_O_2_, and 1.5 *μ*M enzyme in 50 mM sodium phosphate buffer at pH 7.4. The data are fit to one-phase association kinetics.

The increase in fluorescence due to resorufin formation can be used to follow the kinetics of Amplex Red oxidation by the isolated enzymes. The reaction was followed at room temperature and at 65°C to understand how increased temperature influences enzyme activity. As seen in Figure 5, WT CYP119 and N-His-CYP119 showed similar product formation at room temperature, while C-His-CYP119 showed only 20% of WT CYP119 activity. Increasing the temperature to 65°C results in a 80% decrease in the final yield for WT CYP119; however, for N-His-CYP119, this decrease is only 33%, and for C-His-CYP119, almost no decrease in activity is observed. From Figure 5(a), the observed rate constant for the formation resorufin at 25°C was determined to be 6.8 × 10^−3^ s^−1^, 3.7 × 10^−3^ s^−1^, 1.5 × 10^−2^ s^−1^ for WT CYP119, N-His-CYP119, and C-His-CYP119; respectively. Likewise, the observed rate constants for the formation resorufin at 65°C were determined to be 5.2 × 10^−3^ s^−1^, 1.0 × 10^−3^ s^−1^, 1.0 × 10^−2^ s^−1^ for WT CYP119, N-His-CYP119, and C-His-CYP119, respectively (Figure 5(b)). Besides, the rate of product formation does not parallel the yield; indeed, C-His-CYP119 shows the highest rate of product formation but has a significantly lower yield than N-His-CYP119 at 65°C. The molecular mechanism of Amplex Red oxidation by H_2_O_2_ has only been studied for the enzyme horseradish peroxidase; the mechanism of this reaction proved to be extraordinarily complex [19]. Since the detailed mechanism of Amplex Red oxidation catalyzed by CYP119 is not yet known, it is difficult to speculate on the reason for this divergence in rate and yield.

### 3.5. Kinetic Analysis of Amplex Red Oxidation by WT CYP119 and N-His-CYP119

**Figure 6:**
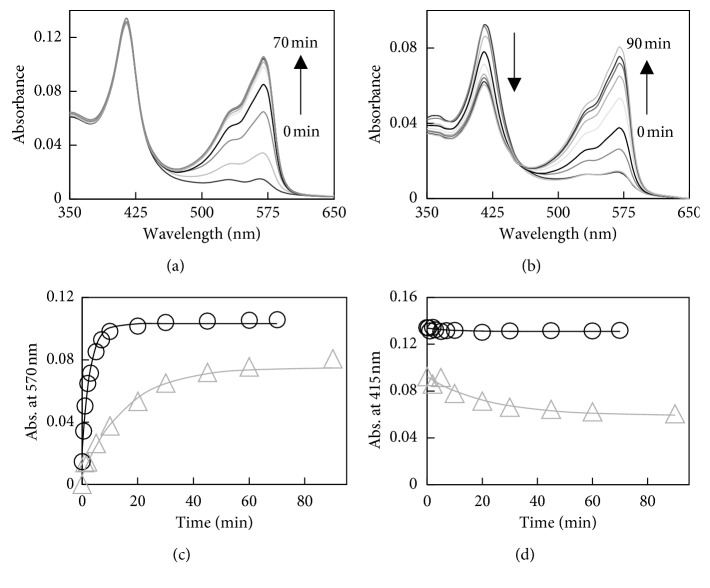
Changes in the UV-Visible spectra during Amplex Red oxidation by H_2_O_2_ in the presence of WT CYP119 (a) and N-His-CYP119 (b); the UV-visible spectra taken at 0, 2, 5, 10, 20, 30, 45, 60, and 90 minutes after addition of H_2_O_2_. The reaction components were 10 *μ*M Amplex Red, 1.5 mM H_2_O_2_, and 1.2 *μ*M enzyme in 50 mM sodium phosphate buffer at pH 7.4. Time course of changes in absorbance at 570 nm (c) and 415 nm (d) in the same reaction (△, N-His-CYP119; ◯, WT CYP119).

For a more detailed analysis of the Amplex Red oxidation, the reaction was followed by UV-Visible spectroscopy. The changes in the UV-Visible spectra during the reaction of 10 *μ*M Amplex Red, 1.5 mM H_2_O_2_, and 1.2 *μ*M WT CYP119 or N-His-CYP119 at 25°C are shown in Figure 6. An increase in 570 nm can be observed during the reaction, this is attributed to resorufin formation. For N-His-CYP119, at the end of the reaction, 16% of Amplex Red was oxidized to product based on the final concentration of resorufin using the extinction coefficient at 570 nm [6]; in comparison, for WT CYP119, 20% of Amplex Red was converted to resorufin. For N-His-CYP119, a concurrent decrease in the Soret absorbance can be observed at 415 nm; a 37% decrease in Soret absorbance is observed by the end of the reaction. On the other hand, WT CYP119 does not show any significant decrease in Soret absorbance during the reaction. The observed rate constant for the formation resorufin was determined to be 5.8 × 10^−3^ s^−1^ and 1.1 × 10^−3^ s^−1^ for WT CYP119 and N-His-CYP119, respectively. The *k*_obs_ for the decay of Soret absorbance for N-His-CYP119 was determined to be 7.6 × 10^−4^ s^−1^.

**Figure 7:**
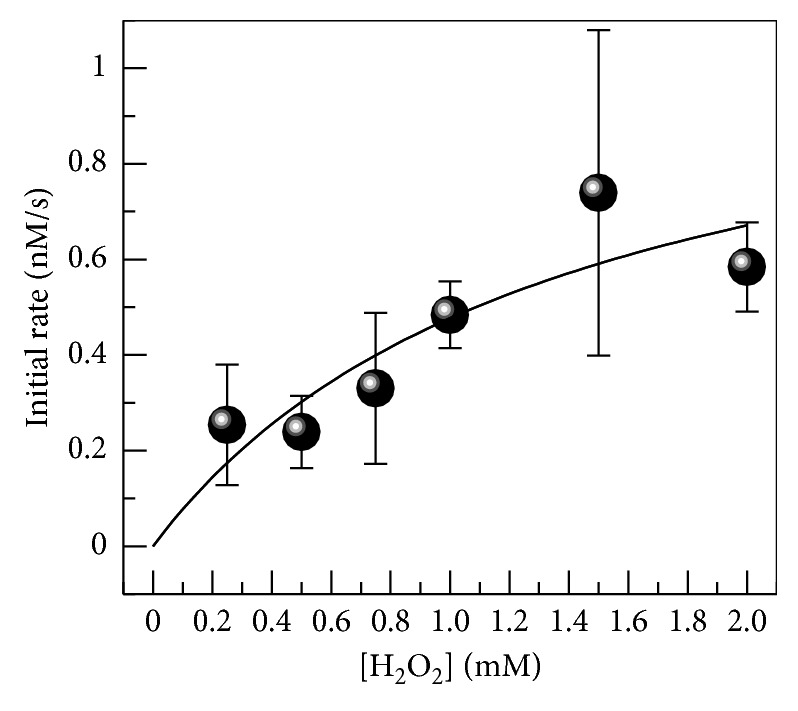
Determination of the kinetic parameters of Amplex Red oxidation by H_2_O_2_ in the presence of N-His-CYP119. Reactions contained 10 *μ*M Amplex Red, 1.5 *μ*M N-His-CYP119, and 0.2–2 mM of H_2_O_2_ in 50 mM sodium phosphate buffer, pH 7.4. Initial rate is obtained from resorufin formation at 30 min.

To determine the kinetic parameters for Amplex Red oxidation by N-His-CYP119, formation of resorufin was followed by absorbance at 570 nm during the reaction of Amplex Red (10 *μ*M) with H_2_O_2_ (0.25–2 mM) in the presence of N-His-CYP119 (1.5 *μ*M). As seen in Figure 6(b), the formation of resorufin increases linearly with time during the first 30 minutes of the reaction; therefore, resorufin concentration at 30 min was used in determination of kinetic parameters. The dependence of initial rate on H_2_O_2_ concentration is shown in Figure 7. Using data at Figure 7, the *K*_M_ was determined to be 1.4 ± 1.1 mM and *k*_cat_ was 7.5 ± 3.2 × 10^−4^ s^−1^ for N-His-CYP119 (in the presence of 10 *μ*M Amplex Red).

## 4. Discussion

Incorporation of polyhistidine tags to proteins is common practice to increase yield and simplify isolation of proteins. While His-tags are commonly far from the active site and relatively small in size, they are not always innocuous. His-tags can lead to changes in the structure and function of the proteins. Here, we studied the effects of C-terminal and N-terminal His-tags on the structure and function of the thermophilic P450 CYP119. We show that the His-tags have distinct influences on structure and function of the CYP119.

C-His-CYP119 and N-His-CYP119 were isolated with high yield and purity using IMAC. The UV-Visible spectra of WT CYP119 were in accordance to previous observations with Soret maximum absorbance at 414 nm and distinct *α*/*β* bands. N-His-CYP119 and C-His-CYP119 showed a similar spectrum to WT CYP119, a side from the 4 nm shift in Soret maximum absorbance to 418 nm (Figure 2).

The effects of His-tags on the thermostability of the enzyme were also investigated by monitoring the UV-Visible spectra and activity of WT CYP119 with increasing temperature. While the maximum Soret absorbance for WT CYP119 did not change significantly with temperature, difference spectra show increased scattering most likely due to aggregation (Figure S4A). Previous studies identified the *T*_m_ for CYP119 as 89°C, and no significant decrease in activity is observed up to 80°C [11, 20]. In this study, the activity of the protein decreased by 80% at 65°C (Figure 5(b)), most likely because the WT CYP119 used is only partially isolated and about 60% pure (Figure S1).

The changes in the UV-Visible spectra were also monitored with increasing temperature for N-His-CYP119. A shift in maximum Soret absorbance of N-His-CYP119 from 418 to 415 nm was observed with increasing temperature (Figure 3(b)). The difference spectra show that the maximum decrease is observed at 425 nm and a subtle increase in absorbance at 390 nm accompanies the decrease in 425 nm (Figure S4B); no significant increase in absorbance due to scattering is observed. In addition, N-His-CYP119 shows high activity at 65°C; therefore, the decrease in Soret absorbance is not due to the loss of heme cofactor but most likely a change in the electronic properties of the heme. Previous studies have shown that a blue shift in Soret maximum absorbance can be attributed to the change in the spin state of the ferric heme iron from low-spin (*S* = 1/2) to high-spin (*S* = 5/2) state [21]. A similar change in UV-Visible spectra is also observed for C-His-CYP119 (Figure 3(c)). The blue shift in Soret absorbance can be more clearly monitored by following the change in the difference in absorbance at 390 nm and 425 nm [∆∆Abs (390–425)] (Figure 3(d)). Previous studies have shown an increase in high-spin state for WT CYP119 with increase in temperature [11]. In accordance with previous results, the ∆∆Abs (390–425) for WT CYP119 increases with temperature. However, as seen in Figure 3(d), N-His-CYP119 shows significantly higher shift in absorbance compared to WT CYP119, showing that the His-tag affected electronic properties of the active site heme.

The changes in Amplex Red oxidation activity with increasing temperature were determined for WT CYP119 and His-tagged variants. These results showed that N-His-CYP119 retained 67% of its activity at 65°C (based on final product formation, Figure 5). No significant decrease in activity was observed for C-His-CYP119 with increasing temperature, but this enzyme only showed 20% activity of WT CYP119 at room temperature. On the other hand, the kinetics of Amplex Red formation at did not show significant changes with increasing temperature.

Since the highest activity and stability were observed for N-His-CYP119, the kinetic parameters for Amplex Red oxidation catalyzed by this protein were investigated. Previous studies have shown that CYP119 can catalyze Amplex Red oxidation [9]; however, kinetic parameters and yield of this reaction have not been investigated. Using the extinction coefficient of resorufin at 570 nm [6], we show that only 16% product formation was observed in the presence of 10 *μ*M Amplex Red, 1.5 mM H_2_O_2_, and 1.5 *μ*M N-His-CYP119 after 3 hours (Figure 6); this corresponds to a single turnover of the enzyme. Therefore, the yield of Amplex Red oxidation is very low. Indeed, when Amplex Red oxidation was monitored in the presence of increasing concentration of H_2_O_2_, the *k*_cat_ obtained was only 7.5 ± 3.2 × 10^−4^ s^−1^. In comparison, previous studies have determined the *k*_cat_ for oxidation of styrene for CYP119 [9] as 1.3 s^−1^. Therefore, the rate of Amplex Red oxidation is significantly lower than that of styrene epoxidation when catalyzed by CYP119.

Comparison of changes in the UV-Visible spectra during the oxidation of Amplex Red by H_2_O_2_ in the presence of WT and N-His-CYP119 can shed light to the changes in enzyme function by the addition of His-tags (Figure 6). Resorufin formation was 5-fold faster in the presence of WT CYP119 compared to N-His-CYP119 at 25°C (*k*_obs_ is 5.8 × 10^−3^ s^−1^ for WT vs 1.1 × 10^−3^ s^−1^ for N-His-CYP119). Similar rate constants were obtained by monitoring the increase in fluorescence at the same temperature; the discrepancy in the rate constant for N-His-CYP119 (3.6 × 10^−3^ s^−1^) is most likely due to large errors observed in the fluorescence results. WT CYP119 has also slightly higher yield than N-His-CYP119 (Figure 6). Furthermore, the Soret absorbance of N-His-CYP119 decreases during the reaction, while WT CYP119 Soret absorbance does not change significantly. Previous studies have shown that treatment of P450s with excess peroxide can result in destruction of porphyrin and apo-enzyme modification, which can be observed as a decrease in Soret absorbance [22–24]. The fact that N-His-CYP119 was more susceptible to heme degradation during Amplex Red oxidation can be due to a more solvent-exposed heme cofactor which is more vulnerable to oxidative modification by H_2_O_2_. Alternatively, the substrate Amplex Red can preclude heme loss by competing with H_2_O_2_ for the reactive heme intermediates formed during the reaction [22]. WT CYP119 can have a higher affinity for Amplex Red which leads to protection from damaging effects of H_2_O_2_. Taken together, the active site of CYP119 showed significant changes by incorporation of an N-terminal His-tag.

## 5. Conclusions

In summary, for CYP119, while His-tags are a very effective strategy in simplifying isolation and purification of the enzyme, they can have unpredictable effects on the heme active site, structure, and activity of the enzymes. Previous studies [3, 9] have used C-terminal His-tagged CYP119; however, there was no direct comparison between the His-tagged protein and WT. In our studies, N-His-CYP119 showed higher stability and activity compared to C-His-CYP119; therefore, it is a good candidate for possible industrial applications. However, care should be taken if N-His-CYP119 is used for mechanistic studies as the electronic structure of the active site seems to have been modified. In addition, the His-tag in N-His-CYP119 is connected to the protein through a thrombin cleavage site; therefore, this construct can be used to obtain tag-free WT CYP119 with additional purification steps. Here, we show that the His-tags can cause structural and functional differences to proteins, while the His-tagged protein still shows expected activity. We suggest that care should be taken in investigation of His-tagged enzymes especially if mechanistic analysis is going to be performed.

## Data Availability

The data used to support the findings of this study are included within the article.

## Supplementary Materials

Supplementary Materials A single supplementary material pdf is available containing the following: the SDS-PAGE analysis of WT and His-tagged variants of CYP119 during the isolation and purification steps and difference absorption spectra of thermostability analysis of WT CYP119 and His-tagged variants.
