# Supplementary material for: Effects of N-Terminal and C-Terminal Polyhistidine Tag on the Stability and Function of the Thermophilic P450 CYP119
Source: Bioinorg Chem Appl. 2019 Jun 20;2019:8080697. doi: 10.1155/2019/8080697 (PMC6610755; doi:10.1155/2019/8080697)
Supplement: Supplementary Materials — A single supplementary material pdf is available containing the following: the SDS-PAGE analysis of WT and His-tagged variants of CYP119 during the isolation and purification steps and difference absorption spectra of thermostability analysis of WT CYP119 and His-tagged variants. [file 8080697.f1.pdf]

Supplementary Material

The effects of N-terminal and C-terminal Polyhistidine Tag on the Stability and Function of the Thermophilic P450 CYP119

Yaprak Aslantas[a] and Nur Basak Surmeli[b*]

[a]*Program in Biotechnology and Bioengineering, İzmir Institute of Technology, Gülbahce, Urla, İzmir, Turkey*

[b]*Department of Bioengineering, İzmir Institute of Technology, Fen Fak. A Blok Oda 205, Gülbahce, Urla Izmir, Turkey*

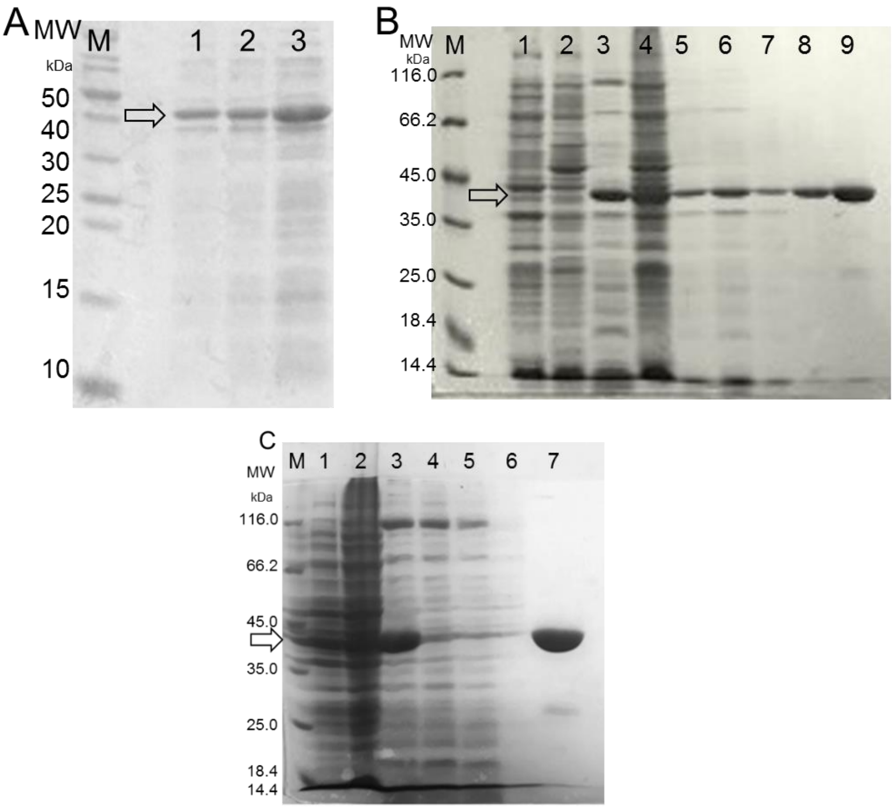

Figure S1. The SDS-PAGE analysis WT CYP119 (A), N-His-CYP119 (B) and C-His-CYP119 (C) during the isolation and purification steps. Lane M, standard proteins marker. Figure S1A: lane 1-3, supernatant of WT CYP119 at different concentration after heat treatment. Figure S1B: lane 1, cell lysate before IPTG and ALA induction, lane 2, cell lysate after IPTG and ALA induction, lane 3, supernatant after heat treatment, lane 4, pellet after heat treatment, lane 5, flow through, lane 6-8, washes, lane 9, purified N-His-CYP119. Figure S1C: lane 1, cell lysate after IPTG and ALA induction, lane 2, pellet after heat treatment, lane 3, supernatant after heat treatment, lane 4, flow through, lane 5-6, washes, lane 7, purified C-His-CYP119. CYP119 is 43 kDa showing the arrows.

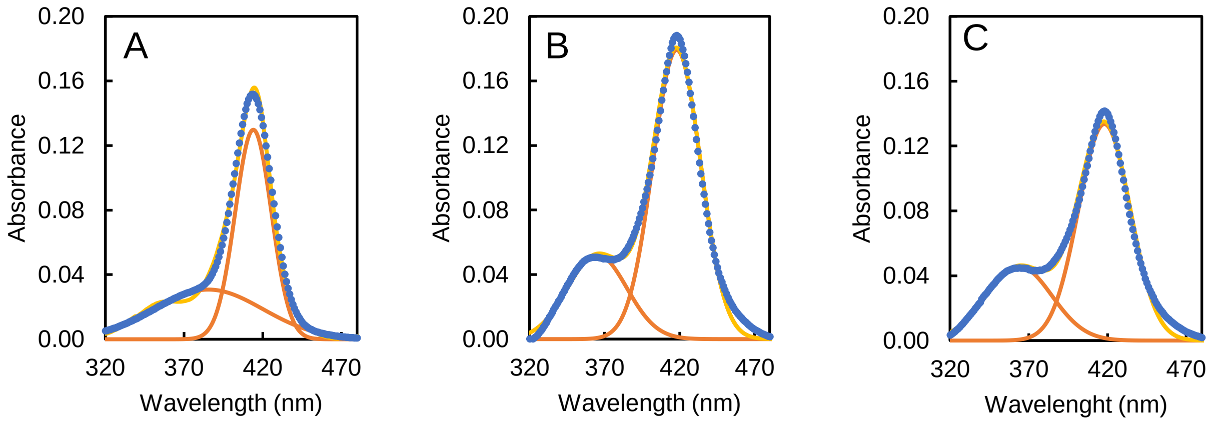

Figure S2. The deconvolution of Soret absorbance of WT CYP119 (A), N-His-CYP119 (B) and C-His-CYP119 (C) after background subtraction. Individual gaussian peaks fitted are shown in orange, the model (obtained from the sum of two peaks) is shown in yellow, the data is shown in blue. The maximum Soret absorbance obtained for WT CYP119 is 414 nm, (δ band 385 nm). The maximum Soret absorbance obtained for N-His-CYP119 is 418 nm, (δ band 365 nm). The maximum Soret absorbance obtained for C-His-CYP119 is 418 nm, (δ band 364 nm).

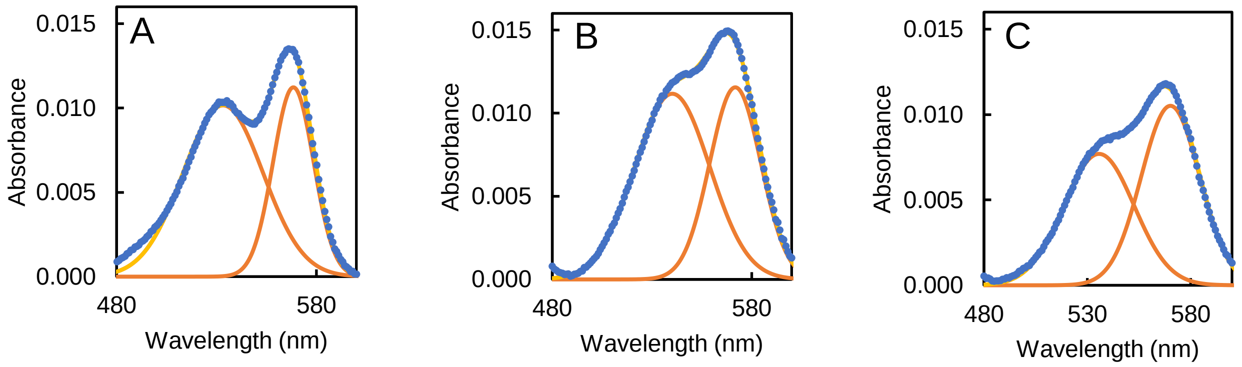

Figure S3. The deconvolution of alpha-beta peaks of WT CYP119 (A), N-His-CYP119 (B) and C-His-CYP119 (C) after background subtraction. Individual gaussian peaks fitted are shown in orange, the model (obtained from the sum of two peaks) is shown in yellow, the data is shown in blue. The α/β peaks obtained for WT are 533 nm and 568 nm. The α/β peaks obtained for N-His-CYP119 are 540 nm and 572 nm. The α/β peaks obtained for C-His-CYP119 are 535 nm and 570 nm.

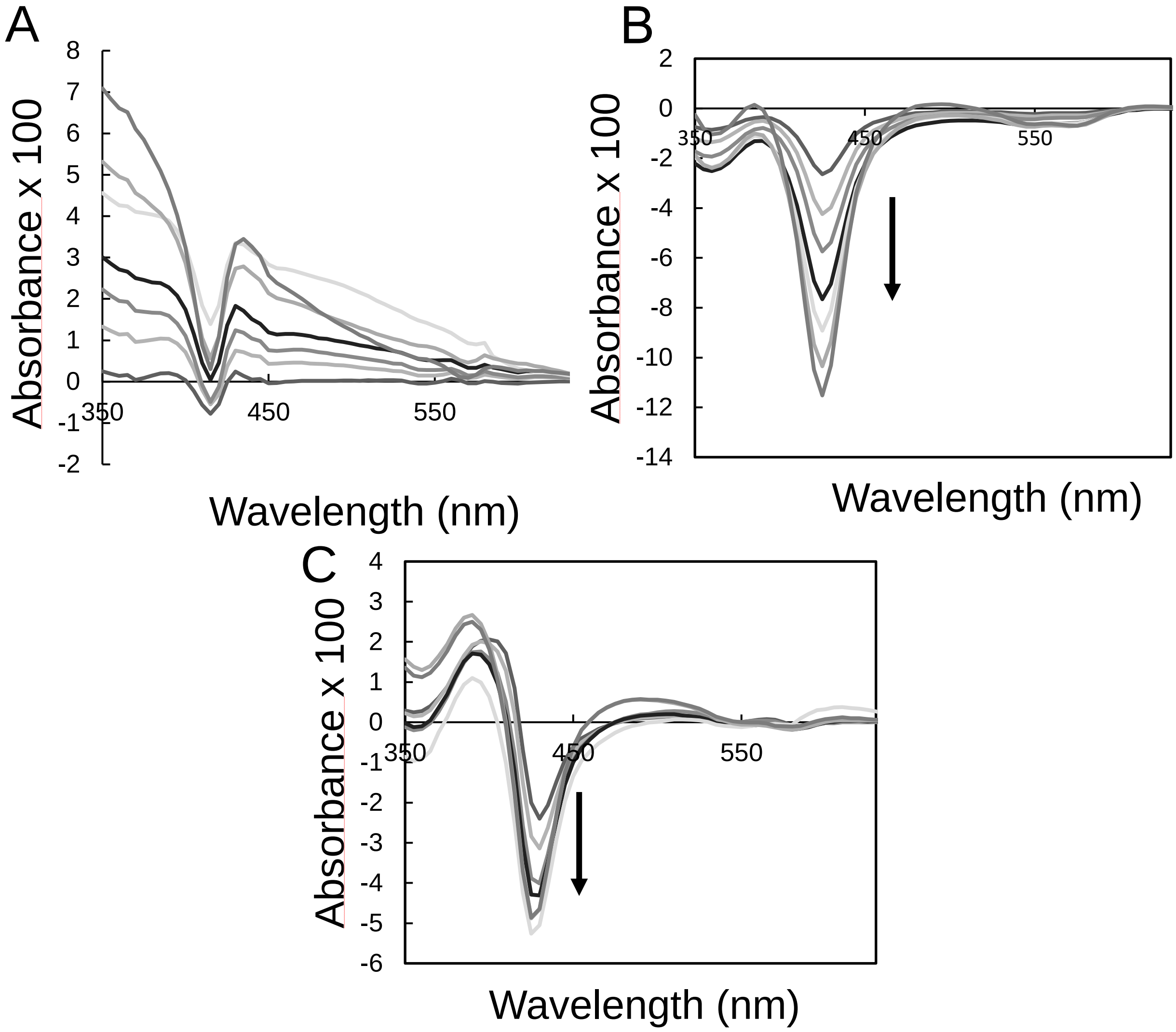

Figure S4. Difference absorption spectra of thermostability analysis of 3.5 µM WT CYP119 (A), 2 µM N-His-CYP119 (B) and 1.4 µM C-His-CYP119 (C). The arrows show Soret decrease of N-His-CYP119 (B) and C- His-CYP119 (C) absorbance with increasing temperature from room temperature to 95 °C.
